# Metabolic Responses to Butyrate Supplementation in LF- and HF-Fed Mice Are Cohort-Dependent and Associated with Changes in Composition and Function of the Gut Microbiota

**DOI:** 10.3390/nu12113524

**Published:** 2020-11-16

**Authors:** Sunhye Lee, Trina A. Knotts, Michael L. Goodson, Mariana Barboza, Elyse Wudeck, Grace England, Helen E. Raybould

**Affiliations:** 1 Department of Anatomy, Physiology, and Cell Biology, University of California Davis School of Veterinary Medicine, Davis, CA 95616, USA; suhlee@ucdavis.edu (S.L.); mlgoodson@ucdavis.edu (M.L.G.); mbarboza@ucdavis.edu (M.B.); evwudeck@gmail.com (E.W.); mgengland@ucdavis.edu (G.E.); 2 Department of Molecular Biosciences, University of California Davis School of Veterinary Medicine, Davis, CA 95616, USA; taknotts@ucdavis.edu; 3 Department of Chemistry, University of California Davis, Davis, CA 95616, USA

**Keywords:** gut microbiota, butyrate, cohort effect, rigor and reproducibility, gut–brain axis, intestinal inflammation, diet-induced obesity

## Abstract

The gut microbiota and associated metabolites have emerged as potential modulators of pathophysiological changes in obesity and related metabolic disorders. Butyrate, a product of bacterial fermentation, has been shown to have beneficial effects in obesity and rodent models of diet-induced obesity. Here, we aimed to determine the beneficial effects of butyrate (as glycerol ester of butyrate monobutyrin, MB) supplementation on metabolic phenotype, intestinal permeability and inflammation, feeding behavior, and the gut microbiota in low-fat (LF)- and high-fat (HF)-fed mice. Two cohorts (separated by 2 weeks) of male C57BL/6J mice (*n* = 24 in each cohort, 6/group/cohort; 6 weeks old) were separated into four weight-matched groups and fed either a LF (10 % fat/kcal) or HF (45% fat/kcal) with or without supplementation of MB (LF/MB or HF/MB) at 0.25% (*w*/*v*) in drinking water for 6 weeks. Metabolic phenotypes (body weight and adiposity), intestinal inflammation, feeding behavior, and fecal microbiome and metabolites were measured. Despite identical genetic and experimental conditions, we found marked differences between cohorts in the response (body weight gain, adiposity, and intestinal permeability) to HF-diet and MB. Notably, the composition of the gut microbiota was significantly different between cohorts, characterized by lower species richness and differential abundance of a large number of taxa, including subtaxa from five phyla, including increased abundance of the genera *Bacteroides*, *Proteobacteria,* and *Parasutterella* in cohort 2 compared to cohort 1. These differences may have contributed to the differential response in intestinal permeability to the HF diet in cohort 2. MB supplementation had no significant effect on metabolic phenotype, but there was a trend to protect from HF-induced impairments in intestinal barrier function in cohort 1 and in sensitivity to cholecystokinin (CCK) in both cohorts. These data support the concept that microbiota composition may have a crucial effect on metabolic responses of a host to dietary interventions and highlight the importance of taking steps to ensure reproducibility in rodent studies.

## 1. Introduction

Obesity has reached epidemic proportions, affecting millions of individuals globally [1]. Obesity is characterized by chronic low-grade inflammation and fat accumulation and is associated with metabolic complications, such as insulin resistance and dyslipidemia [2]. The development of obesity results from a complex interaction of genetic and environmental factors [3]. Increased intake of energy-dense diets, such as high fat and low fiber (HF) diets, is a crucial factor in the development of obesity [4]. Further, recent studies have demonstrated that diet-induced compositional and functional alterations in the gut microbiota play a role in the development and progression of obesity [5]. HF-driven dysbiosis has been linked to chronic low-grade intestinal inflammation and gut barrier dysfunction, promoting translocation of deleterious bacterial metabolites, such as lipopolysaccharides (LPS), into the circulation, resulting in metabolic endotoxemia [6]. Studies have demonstrated that chronic administration of LPS is sufficient to induce hyperphagia, local and systemic inflammation, visceral fat accumulation, and glucose intolerance [6,7]. These findings support a role for the gut microbiota in the development of obesity and related metabolic abnormalities.

The mechanisms by which the gut microbiota contribute to metabolic homeostasis are unclear, but there is evidence that interaction with the host involves bacterial metabolites [8]. Short-chain fatty acids (SCFAs) in particular have been suggested to play an integral role in the regulation of metabolic functions in the host [9]. SCFAs are produced by bacterial fermentation of indigestible fiber; the most abundant metabolites produced in human and mouse gut are acetate, propionate, and butyrate [10]. Increasing attention has been paid to butyrate for its protective role against the pathophysiological processes of obesity [11]. Butyrate has been shown to have a number of different effects on the host including improvement of gut barrier function [12], suppression of the pro-inflammatory response [13,14], and secretion of gut hormones, such as glucagon-like peptide-1 [15], all of which could contribute to alleviation of obesity and related metabolic abnormalities. Butyrate has also been shown to decrease food intake and increase cholecystokinin (CCK)-induced satiety and reduce hepatic fat accumulation; there is evidence to suggest that these actions are mediated via an effect on the gut–brain neural circuit [16].

Although accumulating findings have provided evidence for the metabolic benefits of butyrate, there is limited knowledge on the beneficial effects of luminal butyrate on intestinal permeability and regulation of feeding behavior in the context of obesity. Thus, in the present study, we aimed to investigate the protective effects of chronic butyrate supplementation on HF-induced obesity, hypothesizing that the resulting beneficial effects of butyrate would be associated with compositional and functional changes in the gut microbiota and in bacterial metabolites, reduction in intestinal permeability, and preservation of signaling in the gut–brain axis. To test this hypothesis, mice were fed a low-fat (LF) or HF diet with or without supplementation of monobutyrin (MB) as a glycerol ester of butyrate at 0.25% (*w*/*v*) in drinking water for six weeks, and changes in metabolic phenotypes (body weight and adiposity), intestinal permeability and inflammatory markers, feeding behavior, and fecal microbiome and metabolites were measured.

## 2. Methods and Materials

### 2.1. Animals and Diets

Animals were maintained and handled in accordance with protocols approved by the Institutional Animal Care and Use Committee (University of California, Davis, CA, USA) (18520, Approved Feb-2015). Male C57BL/6J mice (6 weeks old) were purchased from Jackson Laboratory (Sacramento, CA, USA) in two separate cohorts (cohort 1 and cohort 2; *n* = 6/group/cohort), with a 2-week interval for assessment of reproducibility of experimental paradigm. After acclimation for a week, mice were randomly assigned into 4 groups and fed either a low-fat diet (LF/CON; 10% kcal as fat; D12450J; Research Diets, New Brunswick, NJ, USA), a high-fat diet (HF/CON; 45% kcal as fat; D08091803B; Research Diets), or an LF or HF diet with mono- and diglycerides of butyric acid (MB; ProPhorce^TM^ SR 710, Perstorp, Malmö, Sweden; LF/MB or HF/MB) at 0.25% (*w*/*v*) in drinking water for 6 weeks (Table S1). Mice were group-housed (2–4 mice/cage) at 22 °C with 12 h: 12 h light–dark cycles and had ad libitum access to food. Body weight and water intake were measured once a week. At week 5 before feeding behavior testing, fresh fecal samples were collected from each mouse for profiling of microbiota and metabolites. After 6 weeks on the respective treatments, mice were fasted for 6 h and euthanized using pentobarbital (Fatal Plus, Vortech Pharmaceuticals, Dearborn, MI, USA; 300 mg/kg; ip). Blood was collected via cardiocentesis in K3-EDTA tubes and centrifuged at 1000× *g* for 10 min at 4 °C for plasma collection. Visceral fat pads for adiposity assessment and gut tissues for determination of intestinal permeability (see below) were collected at necropsy from *n* = 3/group/cohort. The remaining mice were perfused with paraformaldehyde for obtaining fixed tissue for immunocytochemistry (data not included). Plasma and tissues were snap-frozen and stored at −80 °C until analysis. 

### 2.2. CCK Sensitivity Assessment

After 5 weeks on the respective diets, sensitivity to the satiating effect of CCK was tested. Mice were singly housed and fasted in wire-bottom cages for 6 h during the light phase. CCK (octapeptide, sulfated, Bachem, Torrance, CA, USA, 100 µL at 3 µg/kg; ip) or saline (100 µL; ip) was administered and individual food was placed in the cage and food intake was recorded every 20 min for 60 min.

### 2.3. Fecal Lipocalin-2 Analysis

Fecal homogenates were prepared by adding pre-weighed fecal pellets (1–2) to a 2 mL screwcap tube with 0.1 mm diameter silica beads and 4-mm diameter glass bead and 500 mL fecal extract buffer (phosphate buffered saline + 0.1% Tween-20) and by homogenizing for 40 s at 6.0 M/s at room temperature in the MPBio Fastprep-24 5G homogenizer, and then centrifuging for 5 min at >12,000× *g* at 4 °C. The supernatant was retained for assay. The levels of fecal lipocalin-2 (Lcn-2; R&D Systems, Minneapolis, MN, USA) were detected via enzyme-linked immunosorbent assay (ELISA) according to the manufacturer’s protocol using the homogenate supernatant.

### 2.4. Determination of Intestinal Permeability Ex Vivo

Colon tissue was opened along the mesenteric border, and duplicate samples were mounted in Ussing chambers (Physiologic Instruments, San Diego, CA, USA), exposing 0.1 cm^2^ of tissue surface area to 2.5 ml of oxygenated Krebs-glucose (10 mM) and Krebs-mannitol (10 mM) at 37 °C on the serosal and luminal sides, respectively. The paracellular pathway and transcellular pathway were measured as the flux of FITC-Dextran 4000 (FD-4, Sigma Aldrich, St. Louis, MO, USA) and horseradish peroxidase (HRP Type VI, Sigma Aldrich, St. Louis, MO, USA), respectively. FD-4 (400 µg/mL) and HRP (200µg/mL) were added to the mucosal chamber and samples were collected from the serosal chamber every 30 min for 2 h. Concentration of FD-4 was measured via fluorescence at an excitation of 485 nm and an emission of 538 nm. O-dianisidine substrate was used to detect HRP at an absorbance of 450 nm. Flux was expressed as ng of FD4 or HRP transported per cm^2^ of membrane per hour. Tissue samples with >1.2 × 10^4^ ng/cm^2^/h of FD4 were considered to be damaged and excluded for both HRP and FD4. In addition, tissue replicate samples that varied by more than five-fold or which became damaged while in the Ussing chambers were also excluded. Flux is calculated as the mean of tissues replicates (where available) 60 min after the addition of FD4 and HRP.

#### 2.4.1. Microbiota DNA Sequencing and Analysis

Total DNA was extracted using the MO BIO PowerFecal DNA Kit (Qiagen, Hilden, Germany). Sample libraries were prepared and analyzed by barcoded amplicon sequencing. In brief, the purified DNA was amplified on the V4 region of the 16S rRNA genes via PCR using the following primers: F319 (5′-ACTCCTACGGGAGGCAGCAGT-3′) and R806 (5′-GGACTACNVGGGTWTCTAAT-3′). High-throughput sequencing was performed with Illumina MiSeq paired-end 250-bp run. Raw sequence reads were deposited in the National Center for Biotechnology Information Sequence Read Archive under accession number PRJNA527117. The data derived from sequencing were processed using QIIME2 for 16S-based microbiota analyses [17]. Demultiplexed paired-end sequences that already had barcodes and adapters removed were analyzed using Qiime 2 version 2020.2.0 (qiime2.org). For quality filtering and feature (operational taxonomic unit (OTU)) prediction, we used DADA2 [18]. Forward reads were truncated to 240 nts and reverse reads to 160 nts. Representative sequences were aligned using MAFFT (Multiple Alignment using Fast Fourier Transform) [19]. A phylogenetic tree of the aligned sequences was made using FastTree 2 [20]. OTUs/features were taxonomically classified using a pre-trained Naive Bayes taxonomy classifier. The classifier used was the Silva 132_ 99% OTUs [21]. Tables of taxonomic counts and percentage (relative frequency) were generated. Diversity analyses were run on the resulting OTU/feature.biom tables to provide both phylogenetic and non-phylogenetic metrics of alpha and beta diversity [22]. We obtained a mean of 51,981 ± 7098 (SD) individual sequencing reads per sample (min = 41,700; max= 78,752). After data processing, the average number of sequences for each sample passing through to OTU classification was 37,218 ± 7416 (SD). The average number of OTUs per sample was 105. Alpha (observed species and Faith’s phylogenetic diversity) and beta diversity (phylogenetic UniFrac and non-phylogenetic Bray–Curtis and Jaccard) measures were calculated using the QIIME pipeline (QIIME 2). Rarefaction was used to sample the same number of random reads from each sample for the diversity analyses. The sampling depth was set at 26,200 sequences per sample. Taxon-level abundance data were filtered to remove very low-abundance taxa (<0.05%) and taxa not represented in at least half the samples within a group before further analysis.

#### 2.4.2. Multivariate Analysis of Microbiota and Metadata 

To identify the microbial signatures that are associated with the experimental groups, microbiota genus-level abundance data or metadata were analyzed using partial least squares discriminant analysis (PLS-DA), using packages pls and plsVarSel in R (3.5.1) (cran.r-project.org) Variable importance in projection (VIP) scores were calculated to determine the contribution of the variables to the PLS-DA model. Variables with strong loadings and VIP scores were further evaluated for correlations to experimental metadata. Furthermore, microbiome taxonomic abundance data were analyzed using the linear discriminant analysis (LDA) effect size (LEfSe) method to estimate the effect size of differentially abundant features with biological consistency and statistical significance [23]. Differences in abundances between cohorts were assessed using Kruskal–Wallis test with Dunn’s post hoc test. Kruskal–Wallis pairwise comparisons were used to statistically analyze alpha diversity. Analysis of similarity (ANOSIM) was used to statistically compare groups for beta diversity metrics.

### 2.5. Metabolomic Analysis

Fresh fecal samples were collected at necropsy and stored at −80 °C until analysis. Samples were extracted with nano-pure water (10 mg/mL) at 4 °C with gentle agitation overnight. The homogenates were centrifuged at 21,000× *g* for 5 min, and the supernatants were transferred and centrifuged at 21,000× *g* for additional 20 min. For each fecal sample, aliquots of 20 µL of supernatant were reacted with 20 µL of 200 mM N-(3-Dimethylaminopropyl)-N-ethylcarbodiimide hydrochloride in 5% pyridine and 40 µL of 100 mM 2-nitrophenylhydrazine in 80% acetonitrile /H_2_O (*v*/*v*) with 50 mM HCl. Mixtures were incubated for 30 min at 40 °C and the reaction was terminated by the addition of 400 µL of 10% ACN/H_2_O (*v*/*v*). Samples were centrifuged and transferred into a 96-well injection plate for analysis in an Agilent 6490 triple quadrupole mass spectrometer equipped with the Agilent 1290 infinity LC system (Agilent, Santa Clara, CA,). Chromatographic separations were carried out on an Agilent InfinityLab Poroshell 120 EC-C18 stationary phase (2.1 × 100 mm, 1.9 µm column). Mobile phases were 100% ACN (B) and water with 10% ACN (A). Flow rate was 0.3 mL/min and injection volume was 1 μL. Samples were held at 4 °C in the autosampler, and the column was operated at 40 °C. The mass spectrometer was operated in the multiple reaction monitoring method and positive ionization mode. Parameters were optimized with SCFA standards, and calibration curves were generated for all metabolites measured with corresponding true standards.

### 2.6. Metagenome Inference Using PICRUSt2 and Differential Abundance with ALDEx2

Stand-alone PICRUSt2 was installed and used to run the full pipeline with picrust2_pipeline.py and specifying–per_sequence_contrib as additional parameters using fasta sequences and biom table [24,25]. To examine differences between cohorts, ALDEx2 (ANOVA-like Differential Expression) was used to normalize the PICRUSt2 pathway data by performing log ratio transformation to normalize and Welch’s t and Wilcoxon rank sum tests to statistically test [26,27,28]. A cutoff of Benjamini–Hochberg-adjusted *p* < 0.05 for either was used to identify differentially regulated pathways. A similar approach using ALDEx2 was taken to identify taxa that showed statistically significant divergent populations between cohorts. Principal component and dendogram analyses were used to confirm accurate assignment of samples to the correct cohort. Different taxa (ASVs) contributing to the differentially regulated Metacyc pathways were extracted from PICRUSt2 and visualized using stacked bar charts. Heatmaps, generated with pheatmap in R, were used to visualize the normalized expression value of the predicted pathways for each sample.

### 2.7. Statistical Analysis

Unless stated otherwise, statistical analysis was performed using Prism software (Prism 8.4; GraphPad Software, La Jolla, CA, USA). Data are presented as means ± standard error of the mean (SEM)s. The ROUT test was used to exclude outliers. Two-way analysis of variance (ANOVA) followed by Tukey’s (when the interaction was significant) or Sidak’s (for the main effects) post-hoc test was used to test for differences among groups. Repeated measures 2-way ANOVA was performed for body weight and body weight gain using group assignment (diet x treatment) and time with Sidaks’s post hoc multi-comparisons test. A paired Student’s *t*-test was used to determine statistical significance within groups for CCK sensitivity. Differences were considered significant if *p* < 0.05. Spearman correlations were run using sample metadata and microbiota data using packages Hmisc and corrplot for visualization in R.

## 3. Results

### 3.1. Effects of MB on Body Weight and Adiposity

**Figure 1:**
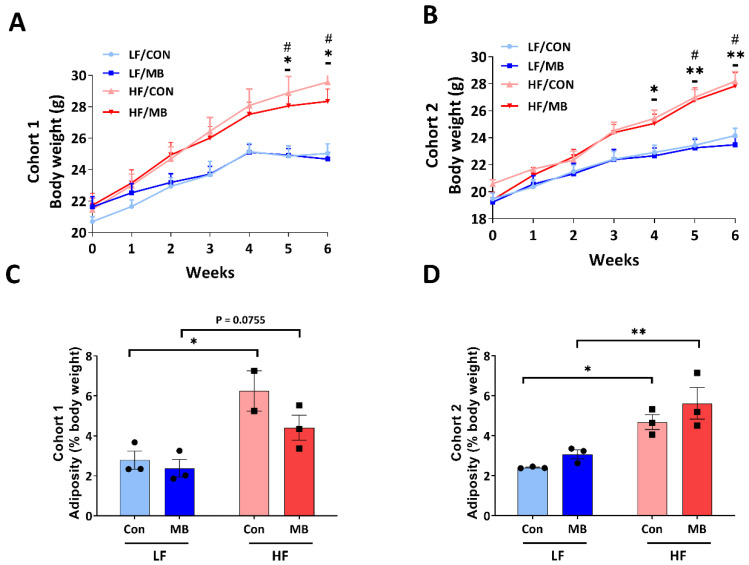
Effect of monobutyrin (MB) supplementation on body weight (**A**,**B**) and adiposity (**C**,**D**) in low-fat (LF) and high-fat (HF) fed mice. Cohort 1 (**A**,**C**) and cohort 2 (**B**,**D**) of mice fed an LF or HF diet with or without 0.25% MB supplementation diet for 6 weeks. Two-way analysis of variance (ANOVA) followed by Tukey’s or Sidak’s post hoc test was used to determine differences among groups. Repeated measures ANOVA was performed for body weight on variable clusters with a random subject effect, whereas variable cluster members, diet, and MB treatment were used as fixed effects. Values are means ± standard of error of mean (SEM)s; *n* = 6 for body weight (gain) and 2–3 for adiposity/group/cohort. Significant differences are denoted as an asterisk (*) for LF/CON vs. HF/CON at * *p* < 0.05 and ** *p* < 0.01 and a sharp (^#^) for LF/MB vs. HF/MB. CON, control; HF, high-fat; HF/CON, HF without MB; HF/MB, HF with 0.25% MB (*w*/*v*) in drinking water; LF, low-fat; LF/CON, LF without MB; LF/MB, LF with 0.25% MB (*w*/*v*) in drinking water; MB, monobutyrin.

In cohort 1 (Figure 1A), from the fourth week of diet treatment and throughout the remaining study period, HF feeding significantly increased body weight compared to LF-fed controls (LF/CON vs. HF/CON, *p* < 0.05 at weeks 5 and 6; LF/MB vs. HF/MB *p* < 0.05 at weeks 5 and 6). In cohort 2 (Figure 1B), HF feeding also resulted in significant increases in body weight compared to LF-fed groups (LF/CON vs. HF/CON and LF/MB vs. HF/MB, *p* < 0.05 at the fourth, fifth, and sixth weeks). MB supplementation had no significant effect on body weight in either LF- or HF-fed mice in either cohort 1 or 2. HF feeding significantly increased visceral adiposity compared to control groups (LF/CON vs. HF/CON, *p* < 0.05) in both cohorts. In cohort 2 (Figure 1F), visceral adiposity was significantly increased by HF feeding in both control and MB-treated mice (LF/CON vs. HF/CON, *p* < 0.05; LF/MB vs. HF/MB, *p* < 0.01).

### 3.2. Effects of MB on Intestinal Inflammation

**Figure 2:**
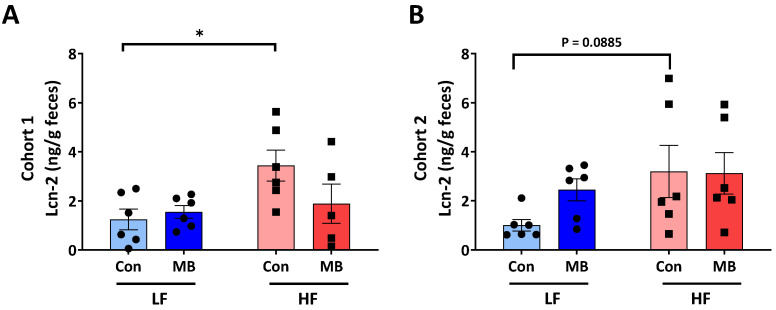
Effect of MB supplementation on fecal lipocalin (Lcn)-2 in cohort 1 (**A**) and cohort 2 (**B**) of mice fed an LF or HF diet with or without 0.25% MB supplementation diet for 6 weeks. Two-way analysis of variance followed by Sidak’s post hoc test was used to determine differences among groups. Values are means ± SEMs; *n* = 6/group/cohort. Asterisk (*) denotes significant differences among groups at * *p* < 0.05. HF, high-fat; HF/CON, HF without MB; HF/MB, HF with 0.25% MB (*w*/*v*) in drinking water; LF, low-fat; LF/CON, LF without MB; LF/MB, LF with 0.25% MB (*w*/*v*) in drinking water; MB, monobutyrin.

Fecal lipocalin-2 (Lcn-2) was measured as a marker of intestinal inflammation [29]. In cohort 1 (Figure 2A), the fecal Lcn-2 level was significantly increased by HF feeding (LF/CON vs. HF/CON, *p* < 0.05). In cohort 2 (Figure 2B), there was a trend for an increase in fecal Lcn-2 in HF/CON mice, but this did not reach statistical significance (LF/CON vs. HF/CON, *p* = 0.0885). There was no significant effect of MB supplementation on fecal Lcn-2 level in either cohort. However, there was a trend for a decrease in fecal Lcn-2 in HF/MB compared to HF/CON mice in cohort 1, but this did not reach statistical significance.

### 3.3. Effects of MB on Intestinal Permeability

**Figure 3:**
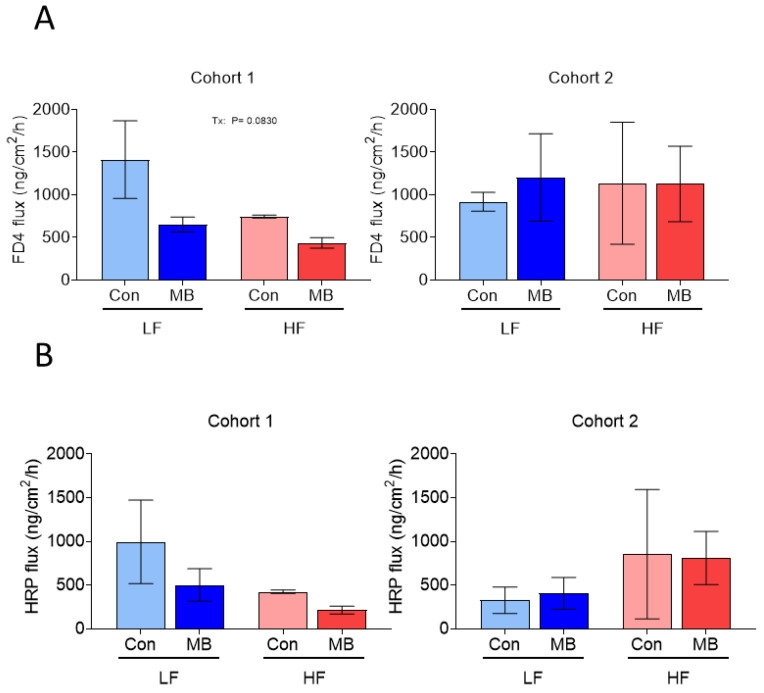
Effect of monobutyrin supplementation on intestinal permeability in mice fed either an LF or HF diet for 6 weeks. (**A**) fluorescein isothiocyanate-dextran (FD)4 flux (paracellular pathway) and (**B)** horseradish peroxidase (HRP) flux (transcellular pathway) in the colon of mice fed either a LF or HF with or without 0.25% MB supplementation for 6 weeks. Two-way analysis of variance followed by Sidak’s post hoc test was used to determine differences among groups. Values are means ± SEMs; *n* = 2–3/group. Significant (*p* < 0.1) main effects are listed on graphs where they occur. FD4, fluorescein isothiocyanate-dextran 4000; HRP, horseradish peroxidase; HF, high-fat diet; LF, low-fat diet; Con, drinking water only control; MB, monobutyrin as 0.25% MB (*w*/*v*) in drinking water.

Intestinal permeability, expressed as paracellular and transcellular permeability, was measured ex vivo in Ussing chambers in colonic tissue collected at necropsy. In cohort 1, the HF diet increased intestinal permeability and there was a trend for a decrease in paracellular permeability in response to MB (Figure 3). In cohort 2, there was no significant effect of diet or treatment on permeability.

### 3.4. Effects of MB on the Integrity of Vagally-Mediated Gut-Brain Signaling

**Figure 4:**
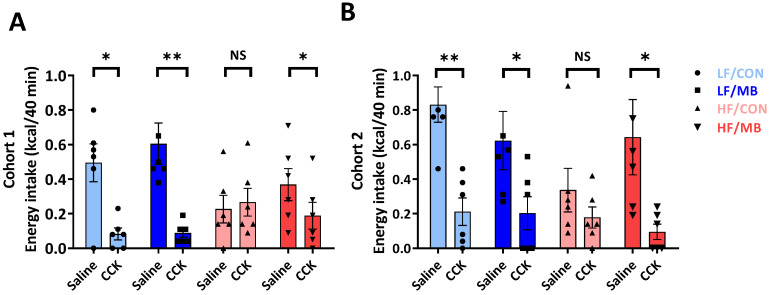
Effect of MB supplementation on vagally-mediated gut–brain signaling. Cholecystokinin (CCK) sensitivity in cohort 1 (**A**) and cohort 2 (**B**) of mice fed an LF or HF diet with or without 0.25% MB supplementation diet for 6 weeks. Paired Student’s t-test was used to determine statistical significance within groups. Values are means ± SEMs; *n* = 6/group/cohort. Asterisk (*) denotes significant differences among groups at * *p* < 0.05 and ** *p* < 0.01. NS, not significant; CCK, cholecystokinin; HF, high-fat; HF/CON, HF without MB; HF/MB, HF with 0.25% MB (*w*/*v*) in drinking water; LF, low-fat; LF/CON, LF without MB; LF/MB, LF with 0.25% MB (*w*/*v*) in drinking water; MB, monobutyrin.

The ability of exogenous CCK to suppress energy intake was determined to measure the effect of MB supplementation on intestinal satiety signaling. In both cohorts 1 and 2 (Figure 4A,B), CCK administration significantly reduced food intake compared to the saline control in both control and MB-treated groups fed an LF diet (cohort 1: CCK vs. saline, *p* < 0.01 LF/MB and *p* < 0.05, LF/CON; cohort 2: CCK vs. saline, *p* = 0.01, LF/CON and *p* < 0.05 LF/MB). CCK had no significant effect on food intake in HF-fed mice (CCK vs. saline, *p* > 0.05, HF/CON) in both cohorts. However, in HF-fed mice given MB, CCK significantly decreased food intake (CCK vs. saline, *p* < 0.05, HF/MB) in both cohorts.

### 3.5. Cohort-Dependent Compositional Changes in the Gut Microbiota

**Figure 5:**
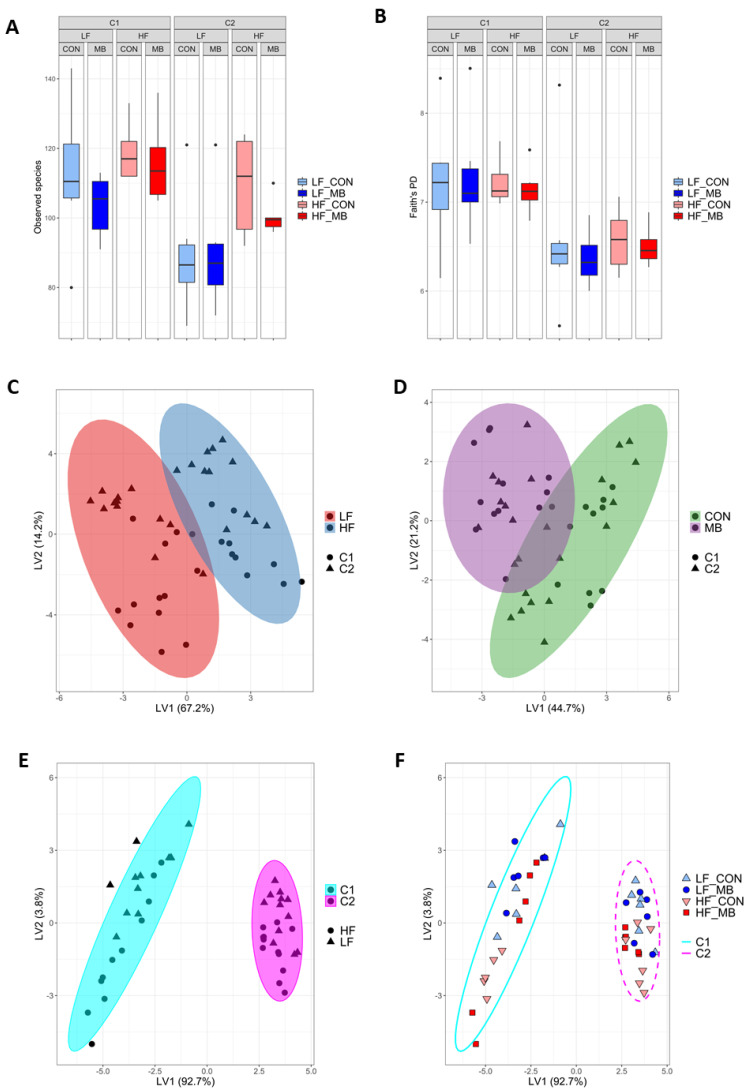
Effect of diet, MB supplementation, and cohort on fecal microbiota diversity and composition. Non-phylogenetic (observed species) (**A**) and phylogenetic (Faith’s phylogenetic diversity) (**B**) alpha diversity measures were calculated for the fecal microbiota. Partial least squares discriminant analysis (PLS-DA) scores plot of genus-level abundance data showing the separation of the variables of diet (**C**), treatment (**D**), and cohort (**E**,**F**). Ellipses represent 95% confidence intervals. Each symbol represents a mouse. Shape and ellipse color indicate group assignments. Latent Variable 1 and 2: LV1 and LV2.

The fecal microbiota was taxonomically classified to profile the bacterial community. Measures of alpha and beta diversity were evaluated (Figure 5). Alpha diversity metrics showed significantly lower biodiversity in cohort 2 for both observed species (Kruskal–Wallis pairwise *p* = 0.002) and Faith’s phylogenetic diversity (*p* < 0.001) (Figure 5A,B). A clear difference between cohorts was also seen in beta diversity for both phylogenetic (UniFrac) and non–phylogenetic (Bray-Curtis or Jaccard) measures of beta diversity using ANOSIM (*p* ≤ 0.002) (Supplemental Table S1). At the genus level, the partial least squares discriminant analysis (PLS-DA) was implemented to characterize the effect of each primary variable: diet (Figure 5C), treatment (Figure 5D), or cohort (Figure 5E,F). Consistent with diversity measures, the separation in the bacterial communities between cohorts was greater than the effect of either diet (LF or HF) or treatment (CON or MB).

To identify differentially abundant microbiota taxa characterizing the cohort effect, several complementary methods, Analysis of the Composition of Microbiomes (ANCOM), ALDEx2, and linear discriminant effect size (LEfSe) were used. There was a strong consensus between the methods. Supplemental Table S2 lists the genera identified by ALDEx2 that are differentially abundant between cohorts, which include members from 2 families within Actinobacteria, 3 families within the Bacteroidetes, 23 taxa within the Firmicutes, 1 genus of Proteobacteria, and 1 class from Tenericutes. The cladogram showing the taxonomical structure between the differential taxa identified by LEfSe is shown in Supplemental Figure S1.

### 3.6. Cohort-Dependent Effects on Microbial Metabolites

**Figure 6:**
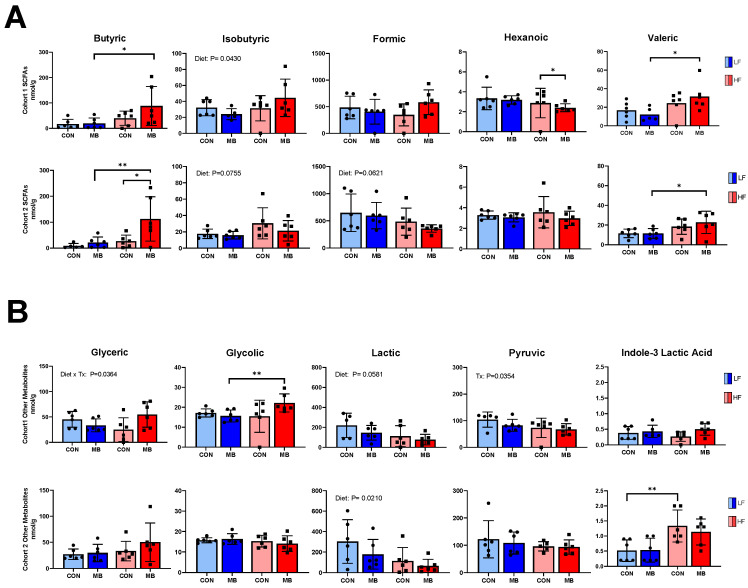
Effect of MB supplementation on fecal microbial metabolites in cohort 1 and cohort 2. (**A**) Fecal short-chain fatty acids and (**B**) other metabolites in mice fed an LF or HF diet with or without 0.25% MB supplementation diet for 6 weeks. Two-way analysis of variance followed by Sidak’s post hoc test was used to determine differences among groups. Values are means ± SEMs; *n* = 6/group/cohort. Asterisk (*) denotes significant differences among groups at * *p* < 0.05, ** *p* < 0.01, NS, not significant; HF, high-fat; HF/CON, HF without MB; HF/MB, HF with 0.25% MB (*w*/*v*) in drinking water; LF, low-fat; LF/CON, LF without MB; LF/MB, LF with 0.25% MB (*w*/*v*) in drinking water; MB, monobutyrin; SCFA, short-chain fatty acid.

In cohort 1 (Figure 6A,B), HF feeding led to significant changes in microbial metabolites compared to the LF control group. In particular, concentrations of butyric, valeric, and glycolic acids were significantly elevated in the HF/MB group compared to the LF/CON group (LF/MB vs. HF/MB, *p* < 0.05 for butyric and valeric acids and *p* < 0.01 for glycolic acid). MB supplementation had no effect on the metabolite profile. In cohort 2, there were significant differences in levels of butyric, valeric, and indole-3-lactic acids between LF- and HF-fed mice (LF/MB vs. HF/MB, *p* < 0.01, <0.05, and <0.01 for butyric, valeric, and indole-3-lactic acids, respectively). There was no significant effect of MB supplementation on most microbial metabolites; however, there was a significant increase in the level of butyric acid in HF-fed groups (HF/CON vs. HF/MB, *p* < 0.05) in cohort 2. In cohort 1, MB treatment significantly reduced hexanoic acid in the HF-fed mice, but this effect was not observed in cohort 2.

The complete list of metabolites measured and the results of 3-way ANOVA showing effect of cohort, treatment and diet is presented in Supplemental Table S3. There were significant cohort differences in isobutyric, valeric, isovaleric, and glycolic acids, and in indole-3-acetic acid.

### 3.7. Metagenome Inference of Pathways Altered between Cohort

**Figure 7:**
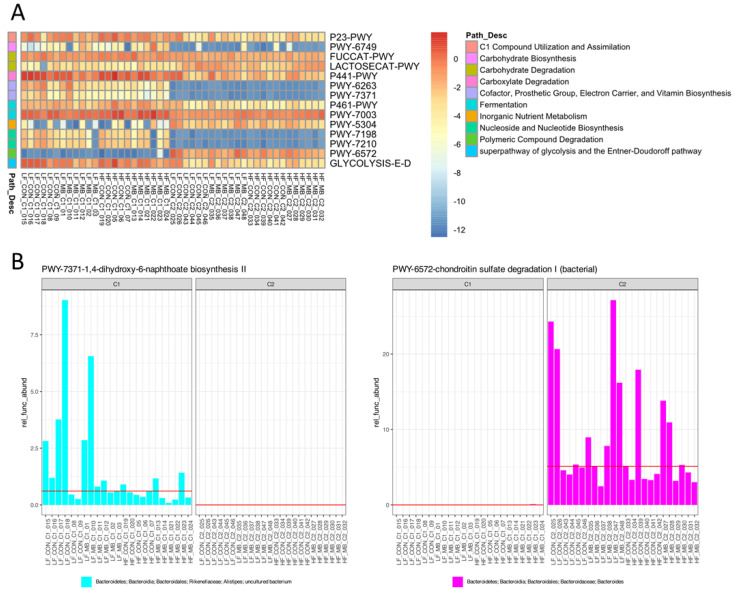
Metacyc pathways identified as differentially abundant between cohorts by metagenome inference using PICRUSt2. (**A**). Heatmap of most highly upregulated and downregulated Metacyc pathways across cohorts selected by Benjamini–Hochberg-adjusted Welch’s t test and Wilcoxon rank sum *p* ≤ 0.05 and effect cutoff of ±1. (**B**). Two pathways that demonstrated unique abundance in cohort 1 (**left**) or cohort 2 (**right**) shown with their contributing taxa.

To understand the potential functional changes in the microbiota between cohorts, we employed PICRUSt2 for metagenome inference using the 16S amplicon sequencing data and ALDEx2 to identify the differentially abundant pathways. We identified 51 Metacyc pathways that differed between cohorts and the contribution of the different taxa to those pathways. The top categories of microbial functions that demonstrated cohort specific regulation included amine and polyamine degradation, carbohydrate biosynthesis and degradation, cofactor, prosthetic group, electron carrier or vitamin synthesis, fermentation, nucleoside and nucleotide biosynthesis, polymeric compound degradation, secondary metabolite degradation, and the tricarboxylic acid cycle (Supplemental Table S4). A higher cutoff was used to identify those pathways with the most robust differential expression based on the metagenome inference (Figure 7). In this set of the 14 most highly regulated predicted pathways, 11 Metacyc pathways (P23-PWY, PWY-6749, FUCCAT-PWY, P441-PWY, PWY- 6263, PWY-7371, P461-PWY, PWY-7003, PWY-7198, PWY-7210, and GLYCOLYSIS-E-D) were decreased and only 3 pathways were increased (LACTOSECAT-PWY, PWY-5304, and PWY-6572) in cohort 2 compared to cohort 1. Important functions for replication and growth, such as pyrimidine deoxyribonucleotide biosynthesis (PWY-7198, PWY-7210), fermentation (PWY-7003), glycolysis (GLYCOLYSIS-E-D), and menaquinone biosynthesis (redox component of electron transfer chain- PWY-6263, PWY-7371)) were reduced in cohort 2. Different populations of bacteria were predicted to contribute to these pathways. In cohort 1, the increased abundance of the genus *Alistipes* in the family Rikenellaceae of the Bacteroidetes phylum contributed to the differential abundance of PWY-7371 (Figure 7B, left panel). In cohort 2, abundance of the genus *Bacteroides* specifically contributed to PWY-6572, demonstrating the unique functional potential of the microbiota in the two cohorts (Figure 7B, right panel).

### 3.8. Correlational and Multivariate Analyses of Metadata and the Microbiota

**Figure 8:**
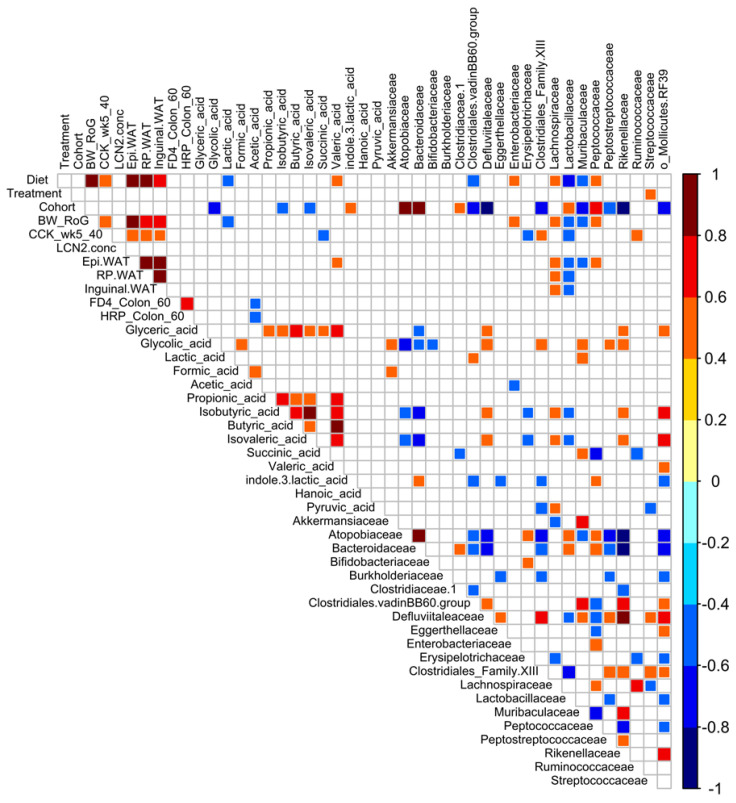
Correlation plot of Spearman’s correlation of project metadata and fecal microbiota. Metadata include body weight rate of gain (BW_RoG), white adipose tissue depot weights (Epidydmal (Epi) and retroperitoneal (RP) WAT), feeding behavior (CCK_wk5_40, CCK food intake at 40 min), intestinal permeability (FD4_colon_60; HRP_colon_60), fecal metabolites, and family-level microbiota. The key experimental variables (diet, treatment, and cohort) are also included for correlation. Colored squares indicate significant correlation. Colored bar at the left provides information about the direction and magnitude of correlation. Uncolored (white) squares demonstrate no significant correlation.

We performed Spearman correlational analyses using a heatmap for visualization to elucidate potential relationships between physiological phenotypes, microbiota, and other metadata in the combined data from both cohorts (Figure 8). As expected, the magnitude of CCK-induced inhibition of food intake was correlated to diet, body weight, and fat pad weight. However, somewhat unexpectedly, the marker of intestinal inflammation, lipocalin-2, did not correlate to any variables. Other intestinal measures, such as permeability in the ileum, similarly did not show strong correlations to diet or treatment. Of all the metadata, only one parameter, the family *Streptococcaceae*, correlated to treatment. The metabolites lactic acid and valeric acid had opposing correlations to diet. Glycolic, isobutyric, isovaleric, and indole 3 lactic acids demonstrated strong correlations to cohort. However, the majority of the correlations were in the microbiota. Unsurprisingly, many microbiota taxa were correlated to diet, but there was a remarkable number of correlations to cohort. When PLS-DA was used to identify the signatures that differentiated the cohorts using all the project metadata, the microbiota dominated the loadings plot and variable importance in projection (VIP) list of the most relevant variables. These approaches highlight the variability in microbiota, even under conditions when mice were housed concurrently in the same room in the vivarium, fed an identical batch/lot of diet, and provided with same preparation of treatment.

## 4. Discussion

In the present study, we investigated the beneficial effects of butyrate supplementation, in the form of MB, on metabolic function, intestinal permeability, and vagally-mediated gut–brain signaling in mice with or without HF-induced metabolic challenge. Overall, there was little effect of MB treatment in either LF- or HF-fed mice on reducing body weight or adiposity. However, MB administration restored CCK-induced inhibition of food intake, suggesting an action of butyrate on the gut–vagal afferent pathway. Previous work has shown that butyrate administration can reduce food intake and body weight gain in HF-fed mice and that the effect on food intake is vagally-mediated [30]. In the present study, mice were not singly housed (to minimize effects of stress), and therefore, we could not measure individual food intake. However, MB administration had no significant effect on the increase in food intake in response to HF feeding measured per cage (three mice per cage) in either cohort (data not shown). The reason for the difference in our findings and the published study is not clear; it is possibly due to the different form of butyrate used in the present study (MB) or to the lack of accurate determination of food intake.

We performed the study in two separate cohorts of mice with a 2-week interval in order to increase the rigor and reproducibility of the data. To this end, we maintained identical environment and experimental conditions for both cohorts; both cohorts were age-matched, purchased from the same vendor, housed in the same room, provided with the same lot of semi-purified LF or HF diets, and exposed to the same animal handlers for daily care, experimental, and euthanasia procedures. However, despite these efforts, there was a significantly different effect between cohorts in the effect of HF diet and MB on intestinal permeability and inflammation. In cohort 1, the HF diet increased intestinal inflammation, as measured by fecal Lcn-2, consistent with our previous observations [31], and there was a trend for MB to decrease Lcn-2. However, there was no significant effect of either HF diet or MB in cohort 2. Similarly, the HF diet increased paracellular intestinal permeability in cohort 1, but not cohort 2, and this was reversed by MB in cohort 1, but not cohort 2. Taken together, these findings suggest a different response to HF diet and MB between cohorts.

The difference in the response of the gut to HF diet and MB in the two cohorts was also seen in a significant difference in the fecal microbiota composition and in its inherent functional capacity and microbial metabolites. HF feeding alters the gut microbiota and promotes intestinal inflammation, which is thought to contribute to the hyperphagic phenotype, weight gain, and other obesity-related metabolic abnormalities [32]. The composition of the fecal microbiota was distinctly different between cohorts, independent of MB supplementation and diet. Specifically, cohort 2 had lower species richness compared to cohort 2. In a study by Hildebrand et al., they investigated the variability of the microbiota of five different lab mouse strains and identified two distinct enterotype-like microbiota communities separated by richness, suggesting the detection of the enterotypes as a common ecological cause that might drive compositional differences in the gut microbiota in mice [33]. As low species richness has been associated with more pronounced overall adiposity, body weight gain, and inflammatory phenotypes in the host [34] as well as the consumption of a high-fat diet [35], the lower species richness observed in cohort 2 may explain the lack of effect of MB on the HF diet-induced changes in the intestine. Further, the fecal microbiota composition of the cohorts was characterized by differential abundance of a large number of genera, with differences across five different phyla. The microbiota of the first cohort was enriched in *Firmcutes* family *Ruminococcaceae* and *Alistipes*, a member of the *Bacteroidetes* phylum. *Alistipes* has been associated with probiotic effects and the SCFAs propionate and acetate, suggesting a protective effect [36,37]. In contrast, one of the taxa that was differentially abundant in cohort 2 was the Proteobacteria *Parasutterella*. Clinical and pre-clinical studies have suggested that an increase in *Proteobacteria* in the gut microbiota serves as a potential indicator of dysbiosis and obesity-related metabolic abnormalities [38,39,40,41]. In particular, the role of *Proteobacteria* has been implicated in the development of gut inflammation [42,43]. For example, Carvalho et al. demonstrated that progression to colitis was closely associated with increased abundance of *Proteobacteria*, especially of the *Escherichia* genus, in TLR5-deficient mice as an inflammation-prone model [43]. Likewise, another study using a colitis-susceptible model showed increased Enterobacteriaceae abundance along with a lowered gut microbiota richness in inflamed mice compared to the healthy control [44]. Metagenome inference comparison of the two cohorts provides evidence for a much more quiescent population in cohort 2 since most of the predicted Metacyc pathways with differential abundance were reduced in cohort 2. An interesting exception, predicted *Bacteroides*-driven PWY-6572, observed as being upregulated in cohort 2, has been reported to be increased in subjects with Crohn’s disease, potentially suggesting a connection to intestinal inflammation or dysfunction [25]. In this regard, the differential abundance of Proteobacteria *Parasutterella* and *Bacteroides* subtaxa may have acted as additional contributors to the increased susceptibility to metabolic alterations in cohort 2.

Along with its direct involvement in the regulation of host metabolism, the gut microbiota also plays a role in host physiology via the production of metabolites. Thus, we determined whether the cohort-dependent differences in the gut microbiota composition led to changes in microbial metabolites. It was not surprising to find little consistency in metabolite profiles between cohorts 1 and 2. However, it is worth noting that both cohorts shared a consistent pattern for fecal butyric acid, even in HF-fed groups with the highest level of butyric acid observed, in the HF/MB group in particular. This consistency in butyric acid may explain the consistent improvement in the feeding response to CCK found in both cohorts. CCK exerts its anorexigenic effect via a vagal-dependent pathway [45]. An HF-driven dysbiotic microbiota and its by-products can alter the innervation of vagal afferent neurons, leading to impairment in CCK-induced satiety signaling [7]. In the present study, while CCK-induced inhibition of food intake was preserved in the LF-fed mice of both cohorts, it was significantly impaired by HF feeding and reversed by MB supplementation, suggesting MB protected the integrity of vagally-mediated pathways. A possible mechanism for MB-induced decrease in food intake is through the anorexigenic effect of butyric acid. As one SCFA, butyrate has been associated with improvements in diet-induced metabolic abnormalities, primarily attributed to a reduction in food intake [16,30]. Notably, a recent study has demonstrated that the anorexigenic effect of exogenous butyrate was attenuated by hepatic branch vagotomy and blunted by chemical denervation of vagal afferents, suggesting that the inhibiting effect of food intake by butyrate depends on the integrity of vagal afferents [30]. The improved response to CCK was associated with higher concentration of fecal butyric acid in the HF/MB-treated mice compared to mice fed an HF diet alone. Given that CCK-induced intestinal satiety signaling was well-preserved against HF feeding in MB-treated mice of cohort 2, despite its profile of the gut microbiota, which was associated with inflammatory states, especially with Proteobacteria known to be toxic to vagal afferent neurons in vitro [41], it is more likely that the increase in butyric acid in cohort 2 was primarily derived from exogenous administration, exerting its anorexigenic action via a vagal-dependent pathway but independently of the fecal microbiota.

One of the most striking findings of the study was the compositional differences in the gut microbiota between the two well-controlled cohorts. While the distinct composition of the gut microbiota in each cohort may have developed during the feeding period of the study, it is more likely that the two cohorts already were harboring distinct microbial communities upon arrival to our animal facility, given the identical experimental conditions and the close clustering of individual microbiota compositions within each cohort. Further, although both cohorts were purchased from the same vendor, each cohort was sourced from a different animal room of the vendor facility. Therefore, the gut microbiota profiles of mice from those specific vivarium rooms could be different. Another possibility is that the cohort difference could have arisen from a litter effect, since mice share a more similar gut microbiota profile to their mother than to that of unrelated mice [46]. In this case, however, the mice from each cohort could not be from the same dams or litters, based on the requested age and time window. While a litter effect is possible, it is unlikely the source of the variation had an effect since several litters would have been needed to provide the *n* = 24 within each cohort. Moreover, it is also possible that this temporal difference was associated with the shipment group effect, exposing mice to different environments of their time window, thus increasing their susceptibility to gut microbiota divergence [46]. Consequently, these likely differences in preexisting gut microbial composition, or baseline microbiota composition, may have modulated the metabolic responses to MB supplementation in each cohort as previously reported [47,48], which possibly explains discrepant outcomes between cohorts of this study.

## 5. Conclusions

Further research is needed to better understand how and how much deterministic factors can cause variation in baseline microbiota composition and how this may or may not have a causal effect on the reproducibility and translation of preclinical findings.

## Supplementary Materials

The following are available online at https://www.mdpi.com/2072-6643/12/11/3524/s1, Supplemental Figure S1: Cladogram generated from LEfSe analysis showing the most differentially abundant taxa enriched in microbiota. Supplemental Table S1: Beta diversity Metrics: ANOSIM pairwise comparisons by diet, treatment, and cohort. Supplemental Table S2: Differentially abundant fecal microbiota genera between cohorts. Supplemental Table S3: Effect of diet, treatment, and cohort on levels of bacterial metabolites in feces. Supplemental Table S4: Differentially abundant PICRUSt2 identified Metacyc pathways between cohorts.
