# Supplementary material for: Metabolic Responses to Butyrate Supplementation in LF- and HF-Fed Mice Are Cohort-Dependent and Associated with Changes in Composition and Function of the Gut Microbiota
Source: Nutrients. 2020 Nov 16;12(11):3524. doi: 10.3390/nu12113524 (PMC7696936; doi:10.3390/nu12113524)
Supplement: Supplementary file 1 [file nutrients-12-03524-s001.pdf]

Supplemental Figure 1

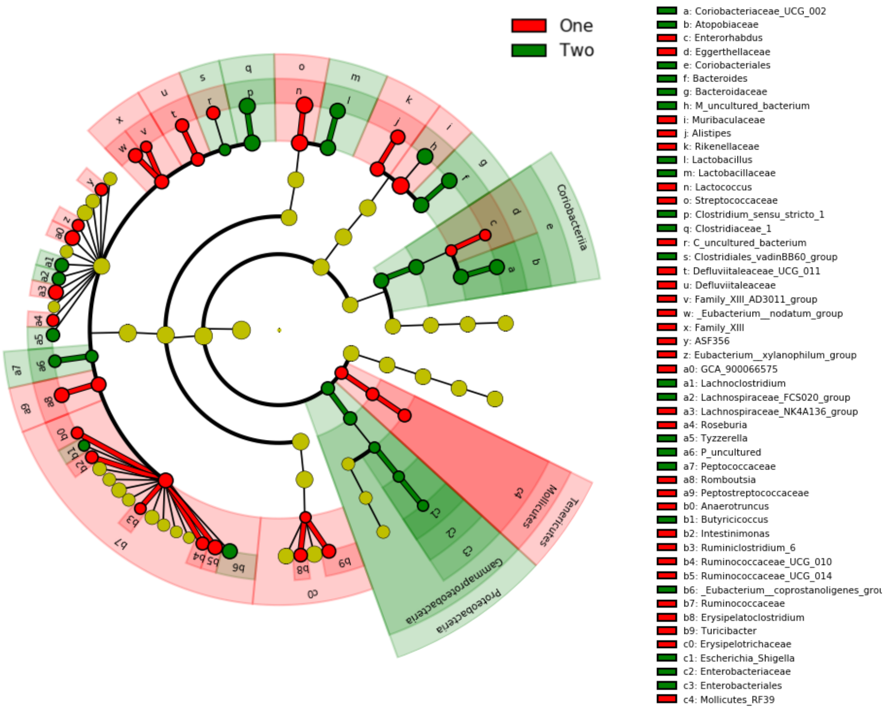

Supplemental Figure 1: Cladogram generated from LEfSe analysis showing the most differentially abundant taxa enriched in microbiota; red indicating cohort 1 and green cohort 2.

Supplemental Table 1: Beta diversity Metrics: ANOSIM pairwise comparisons by diet, treatment, and cohort.

**Variable**	**Group 1**	**Group 2**	**Unweighted UF**	**Weighted UF**	**Bray-Curtis**	**Jaccard**
Diet	LF	HF	0.035	0.003	0.001	0.015
Tx	CON	MB	0.626	0.044	0.078	0.322
Cohort	C1	C2	0.001	0.002	0.001	0.001

Supplemental Table 2: Differentially abundant fecal microbiota genera between cohorts. Magnitude and directionality demonstrated by effect parameter (positive effect- increased in cohort 2; negative effect- decreased in cohort 2.) A cutoff of Benjamini-Hochberg adjusted $p < 0.05$ for Welch's t or Wilcoxon rank sum tests were used to identify differentially regulated genera. (n=23-24/cohort)

Taxa	effect	Welch.BH	Wilcox.BH
Bacteroidetes;Bacteroidia;Bacteroidales;Bacteroidaceae;Bacteroides	4.300	0.0000	0.0000
Firmicutes;Clostridia;Clostridiales;Ruminococcaceae;[Eubacterium] coprostanoligenes group	4.150	0.0000	0.0000
Actinobacteria;Coriobacteriia;Coriobacteriales;Atopobiaceae;Coriobacteriaceae UCG-002	3.799	0.0000	0.0000
Firmicutes;Clostridia;Clostridiales;Ruminococcaceae;Ruminococcaceae UCG-010	-3.042	0.0000	0.0000
Bacteroidetes;Bacteroidia;Bacteroidales;Rikenellaceae;Alistipes	-2.880	0.0000	0.0000
Firmicutes;Clostridia;Clostridiales;Lachnospiraceae;Roseburia	-1.954	0.0000	0.0000
Firmicutes;Clostridia;Clostridiales;Lachnospiraceae;GCA-900066575	-1.945	0.0000	0.0000
Bacteroidetes;Bacteroidia;Bacteroidales;Muribaculaceae;uncultured bacterium	1.892	0.0000	0.0000
Firmicutes;Clostridia;Clostridiales;Lachnospiraceae;Lachnospiraceae FCS020 group	1.716	0.0000	0.0000
Firmicutes;Clostridia;Clostridiales;Defluviitaleaceae;Defluviitaleaceae UCG-011	-1.384	0.0000	0.0000
Actinobacteria;Coriobacteriia;Coriobacteriales;Eggerthellaceae;Enterorhabdus	-1.206	0.0001	0.0000
Firmicutes;Clostridia;Clostridiales;Lachnospiraceae;ASF356	-1.142	0.0002	0.0000
Firmicutes;Clostridia;Clostridiales;Clostridiales vadinBB60 group;uncultured bacterium	-1.088	0.0001	0.0000
Firmicutes;Clostridia;Clostridiales;Family XIII;[Eubacterium] nodatum group	-1.041	0.0003	0.0000
Firmicutes;Clostridia;Clostridiales;Peptococcaceae;uncultured	0.974	0.0151	0.0000
Firmicutes;Clostridia;Clostridiales;Ruminococcaceae;Intestinimonas	-0.926	0.0022	0.0002
Bacteroidetes;Bacteroidia;Bacteroidales;Muribaculaceae;__	-0.855	0.0002	0.0003
Firmicutes;Bacilli;Lactobacillales;Lactobacillaceae;Lactobacillus	0.774	0.0010	0.0016
Firmicutes;Clostridia;Clostridiales;Lachnospiraceae;Lachnoclostridium	0.694	0.0042	0.0058
Firmicutes;Clostridia;Clostridiales;Ruminococcaceae;Ruminococcaceae UCG-014	-0.694	0.0030	0.0007
Firmicutes;Clostridia;Clostridiales;Lachnospiraceae;[Eubacterium] xylanophilum group	-0.687	0.0203	0.0154
Firmicutes;Erysipelotrichia;Erysipelotrichales;Erysipelotrichaceae;Erysipelatoclostridium	-0.668	0.0083	0.0045
Firmicutes;Clostridia;Clostridiales;Ruminococcaceae;Butyricicoccus	0.664	0.0177	0.0091
Firmicutes;Clostridia;Clostridiales;Family XIII;Family XIII AD3011 group	-0.602	0.0395	0.0308
Firmicutes;Clostridia;Clostridiales;Ruminococcaceae;Ruminiclostridium 9	0.591	0.0083	0.0137
Firmicutes;Erysipelotrichia;Erysipelotrichales;Erysipelotrichaceae;Turicibacter	-0.585	0.0368	0.0608
Firmicutes;Clostridia;Clostridiales;Clostridiales vadinBB60 group;__	0.581	0.0235	0.0173
Tenericutes;Mollicutes;Mollicutes RF39;__;__	-0.571	0.0069	0.0016
Proteobacteria;Gammaproteobacteria;Betaproteobacteriales;Burkholderiaceae;Parasutterella	0.555	0.0067	0.0570
Firmicutes;Clostridia;Clostridiales;Clostridiaceae 1;Clostridium sensu stricto 1	0.521	0.0033	0.0064
Firmicutes;Clostridia;Clostridiales;Peptostreptococcaceae;Romboutsia	-0.505	0.0081	0.0043

Supplemental Table 3: Effect of diet, treatment, and cohort on levels of bacterial metabolites in feces. Data expressed as nmol/g, mean ± SEM n=6 for each group.

	Cohort1				Cohort2				3 way ANOVA P values						
**Variable**	**LF_CON**	**LF_MB**	**HF_CON**	**HF_MB**	**LF_CON**	**LF_MB**	**HF_CON**	**HF_MB**	**Cohort**	**Diet**	**Treatment**	**Cohort x Diet**	**Cohort x Treatment**	**Diet x Treatment**	**Cohort x Diet x Treatment**
**Acetic**	**858.04±44.39**	**949.62±148.98**	**813.02±43.02**	**922.15±170.15**	**1267.48±292.7**	**1047.23±227.37**	**949.52±110.13**	**761.72±83.52**	**0.307**	**0.155**	**0.659**	**0.262**	**0.200**	**0.915**	**0.975**
**Butyric**	**16.87±7.54**	**19.47±8.66**	**47.62±10.13**	**88.23±31.44**	**9.06±4.02**	**22.08±8.54**	**27.08±9.48**	**112.63±34.89**	**0.980**	**0.000**	**0.012**	**0.866**	**0.306**	**0.045**	**0.522**
**Isobutyric**	**32.1±4.11**	**23.98±3.07**	**37.68±1.88**	**44.43±9.56**	**17.57±2.37**	**15.9±1.88**	**30.43±7.77**	**21.37±5.12**	**0.001**	**0.006**	**0.435**	**0.618**	**0.545**	**0.629**	**0.154**
**Formic**	**486.1±86.71**	**487.7±60.56**	**415.88±58.88**	**584.38±94.98**	**647.88±140.92**	**596.35±98.52**	**485.12±101.82**	**360.43±27.81**	**0.661**	**0.163**	**0.982**	**0.113**	**0.194**	**0.722**	**0.365**
**Hexanoic**	**3.33±0.46**	**3.18±0.17**	**3.44±0.23**	**2.4±0.16**	**3.28±0.16**	**3.07±0.18**	**3.57±0.62**	**2.97±0.28**	**0.575**	**0.599**	**0.038**	**0.362**	**0.691**	**0.180**	**0.590**
**Propionic**	**102.13±18.13**	**79.35±17.62**	**81.86±20.55**	**143.98±40.54**	**88.77±5.67**	**81.47±7.77**	**92.88±16.08**	**98.08±28.89**	**0.467**	**0.306**	**0.557**	**0.709**	**0.513**	**0.129**	**0.256**
**Valeric**	**16.75±3.71**	**12.18±3.22**	**29.18±2.19**	**31.48±6.19**	**11.52±1.79**	**11.47±2.03**	**18.43±3.19**	**22.75±4.63**	**0.021**	**<0.0001**	**0.851**	**0.208**	**0.540**	**0.294**	**0.814**
**Isovaleric**	**30.98±4.81**	**30.83±5.54**	**35.18±2.1**	**33.95±3.64**	**17.82±1.94**	**16.55±1.37**	**24.85±2.72**	**19.23±4.8**	**<0.0001**	**0.115**	**0.439**	**0.821**	**0.605**	**0.610**	**0.759**
**Glyceric**	**45.27±6.35**	**33.43±5.2**	**29.96±10.02**	**54.78±10.27**	**27.08±4.06**	**29.52±6.73**	**33.13±7.64**	**50.13±15.09**	**0.349**	**0.196**	**0.200**	**0.412**	**0.797**	**0.046**	**0.380**
**Glycolic**	**17.17±0.83**	**15.65±1.23**	**18.62±1.35**	**22.2±1.86**	**15.72±0.5**	**16.35±1.06**	**15.27±1.16**	**14.08±1.57**	**0.001**	**0.146**	**0.673**	**0.005**	**0.467**	**0.363**	**0.059**
**Lactic**	**220±54.76**	**145.8±30.11**	**134.2±45.34**	**77.67±21.39**	**303.88±86.69**	**179.03±58.93**	**115.77±52.2**	**66.22±26.57**	**0.552**	**0.003**	**0.043**	**0.319**	**0.766**	**0.527**	**0.694**
**Pyruvic**	**104.08±12.79**	**82.67±9.19**	**87.72±2.61**	**67.55±8.68**	**122.13±27.76**	**108.47±16.44**	**95.88±7.74**	**93.87±10.63**	**0.066**	**0.088**	**0.174**	**0.822**	**0.534**	**0.757**	**0.802**
**Succinic**	**18.37±3.03**	**19.85±2.94**	**22.36±4.74**	**16.97±3.08**	**19.9±5.36**	**13.78±3.08**	**12.2±1.59**	**17.2±3.03**	**0.150**	**0.749**	**0.613**	**0.587**	**0.778**	**0.669**	**0.075**
**Indole-3 LA**	**0.38±0.08**	**0.43±0.08**	**0.32±0.04**	**0.5±0.08**	**0.52±0.14**	**0.53±0.16**	**1.33±0.22**	**1.13±0.18**	**<0.0001**	**0.001**	**0.906**	**0.001**	**0.297**	**0.826**	**0.381**

Supplemental Table 4: Differentially abundant PICRUSt2 identified Metacyc pathways between cohorts. Magnitude and directionality demonstrated by effect parameter (positive effect- increased in cohort 2; negative effect- decreased in cohort 2.) A cutoff of Benjamini-Hochberg adjusted $p < 0.05$ for Welch's t (we.eBH)or Wilcoxon rank sum (wi.eBH) tests were used to identify differentially regulated pathways. Specific pathway description and general category description of pathways are provided for each pathway. (n=23-24/cohort)

Pathway	effect	we.eBH	wi.eBH	Path Description	Path Category
PWY-6572	6.230	4.47E-17	1.22E-11	chondroitin sulfate degradation I (bacterial)	Polymeric Compound Degradation
PWY-6263	-4.314	1.32E-20	1.22E-11	superpathway of menaquinol-8 biosynthesis II	Cofactor, Prosthetic Group, Electron Carrier, and Vitamin Biosynthesis
PWY-7210	-3.947	1.71E-09	2.92E-07	pyrimidine deoxyribonucleotides biosynthesis from CTP	Nucleoside and Nucleotide Biosynthesis
PWY-7198	-3.791	1.06E-09	2.86E-07	pyrimidine deoxyribonucleotides de novo biosynthesis IV	Nucleoside and Nucleotide Biosynthesis
PWY-7371	-3.410	6.58E-18	1.22E-11	1,4-dihydroxy-6-naphthoate biosynthesis II	Cofactor, Prosthetic Group, Electron Carrier, and Vitamin Biosynthesis
P441-PWY	-1.541	3.26E-08	4.82E-07	superpathway of N-acetylneuraminate degradation	Carboxylate Degradation
GLYCOLYSIS-E-D	-1.527	3.75E-08	4.75E-07	superpathway of glycolysis and the Entner-Doudoroff pathway	superpathway of glycolysis and the Entner-Doudoroff pathway
PWY-5304	1.510	1.21E-06	5.38E-09	superpathway of sulfur oxidation (Acidianus ambivalens)	Inorganic Nutrient Metabolism
P461-PWY	-1.503	8.83E-08	7.39E-07	hexitol fermentation to lactate, formate, ethanol and acetate	Fermentation
PWY-6749	-1.498	1.13E-06	5.84E-06	CMP-legionaminate biosynthesis I	Carbohydrate Biosynthesis
PWY-7003	-1.361	1.80E-07	3.25E-07	glycerol degradation to butanol	Fermentation
P23-PWY	-1.334	1.79E-06	1.06E-05	reductive TCA cycle I	C1 Compound Utilization and Assimilation
FUCCAT-PWY	-1.325	1.33E-06	2.73E-06	fucose degradation	Carbohydrate Degradation
LACTOSECAT-PWY	1.322	3.15E-04	7.45E-07	lactose and galactose degradation I	Carbohydrate Degradation
P125-PWY	-0.993	8.88E-05	2.79E-06	superpathway of (R,R)-butanediol biosynthesis	Fermentation
GLUCUROCAT-PWY	-0.979	1.44E-04	5.24E-04	superpathway of beta-D-glucuronosides degradation	Secondary Metabolite Degradation
PWY-6396	-0.975	4.40E-04	2.07E-06	superpathway of 2,3-butanediol biosynthesis	Fermentation
P163-PWY	-0.916	3.23E-04	2.73E-04	L-lysine fermentation to acetate and butanoate	Fermentation
GALACT-GLUCUROCAT-PWY	-0.847	6.67E-04	1.42E-03	superpathway of hexuronide and hexuronate degradation	superpathway of hexuronide and hexuronate degradation
RHAMCAT-PWY	0.844	4.35E-04	3.62E-04	L-rhamnose degradation I	Carbohydrate Degradation
GLUCARDEG-PWY	-0.814	3.42E-03	6.50E-04	D-glucarate degradation I	Secondary Metabolite Degradation
NAD-BIOSYNTHESIS-II	-0.803	1.38E-03	1.69E-03	NAD salvage pathway III (to nicotinamide riboside)	Cofactor, Prosthetic Group, Electron Carrier, and Vitamin Biosynthesis
PWY0-41	-0.770	8.75E-03	2.62E-02	allantoin degradation IV (anaerobic)	Amine and Polyamine Degradation
PWY0-1533	0.697	1.90E-02	6.99E-03	methylphosphonate degradation I	Inorganic Nutrient Metabolism
METH-ACETATE-PWY	-0.696	9.26E-03	3.53E-03	methanogenesis from acetate	Respiration
HEXITOLDEGSUPER-PWY	-0.691	1.16E-02	8.45E-03	superpathway of hexitol degradation (bacteria)	Secondary Metabolite Degradation
PWY-5705	-0.689	1.31E-02	4.29E-03	allantoin degradation to glyoxylate III	Amine and Polyamine Degradation
GLUCARGALACTSUPER-PWY	0.676	2.14E-02	9.88E-03	superpathway of D-glucarate and D-galactarate degradation	superpathway of D-glucarate and D-galactarate degradation
PWY-7237	0.675	1.04E-02	1.48E-02	myo-, chiro- and scyllo-inositol degradation	Secondary Metabolite Degradation
PWY-6478	-0.665	9.24E-03	1.96E-02	GDP-D-glycero-alpha-D-manno-heptose biosynthesis	Carbohydrate Biosynthesis
GALACTARDEG-PWY	0.654	2.16E-02	9.37E-03	D-galactarate degradation I	Secondary Metabolite Degradation
HEMESYN2-PWY	-0.641	1.41E-02	1.56E-02	heme b biosynthesis II (anaerobic)	Cofactor, Prosthetic Group, Electron Carrier, and Vitamin Biosynthesis
P162-PWY	-0.634	3.82E-03	3.78E-04	L-glutamate degradation V (via hydroxyglutarate)	Fermentation
PWY-6895	-0.626	1.96E-02	7.26E-04	superpathway of thiamine diphosphate biosynthesis II	Cofactor, Prosthetic Group, Electron Carrier, and Vitamin Biosynthesis
GALACTUROCAT-PWY	-0.626	1.25E-02	1.41E-02	D-galacturonate degradation I	Secondary Metabolite Degradation
PWY-6891	-0.622	1.11E-02	3.23E-03	thiazole biosynthesis II (aerobic bacteria)	Cofactor, Prosthetic Group, Electron Carrier, and Vitamin Biosynthesis
PWY-5913	0.597	3.63E-02	1.70E-02	partial TCA cycle (obligate autotrophs)	TCA cycle
PWY-7254	-0.589	2.57E-02	5.95E-03	TCA cycle VII (acetate-producers)	TCA cycle
PWY-5022	-0.586	7.34E-03	3.87E-03	4-aminobutanoate degradation V	Fermentation
PWY-5837	-0.581	1.79E-02	1.84E-02	2-carboxy-1,4-naphthoquinol biosynthesis	Cofactor, Prosthetic Group, Electron Carrier, and Vitamin Biosynthesis
PWY-5863	-0.580	1.76E-02	1.77E-02	superpathway of phylloquinol biosynthesis	Cofactor, Prosthetic Group, Electron Carrier, and Vitamin Biosynthesis
PWY-7242	-0.579	1.70E-02	1.73E-02	D-fructuronate degradation	Secondary Metabolite Degradation
PWY-6471	0.573	2.42E-02	3.85E-02	peptidoglycan biosynthesis IV (Enterococcus faecium)	Cell Structure Biosynthesis
PWY-7456	0.569	1.08E-02	1.35E-03	beta-(1,4)-mannan degradation	Polymeric Compound Degradation
PWY0-166	0.558	9.30E-03	2.51E-02	superpathway of pyrimidine deoxyribonucleotides de novo biosynthesis (E. coli)	Nucleoside and Nucleotide Biosynthesis
PWY-5005	-0.546	1.98E-02	4.03E-03	biotin biosynthesis II	Cofactor, Prosthetic Group, Electron Carrier, and Vitamin Biosynthesis
P164-PWY	-0.523	3.07E-02	4.40E-02	purine nucleobases degradation I (anaerobic)	Nucleoside and Nucleotide Degradation
REDCITCYC	-0.520	2.46E-02	1.60E-02	TCA cycle VIII (helicobacter)	TCA cycle
PWY-5677	-0.486	2.27E-02	1.29E-02	succinate fermentation to butanoate	Fermentation
PWY-6876	0.450	1.21E-02	2.82E-02	isopropanol biosynthesis (engineered)	isopropanol biosynthesis (engineered)
PWY0-1261	0.369	2.40E-02	3.65E-02	anhydromuropeptides recycling I	Secondary Metabolite Degradation
